# Preferential Accumulation of ^14^C-*N*-Glycolylneuraminic Acid over ^14^C-*N*-Acetylneuraminic Acid in the Rat Brain after Tail Vein Injection

**DOI:** 10.1371/journal.pone.0131061

**Published:** 2015-06-22

**Authors:** Risa Taguchi, Akira Minami, Yukino Matsuda, Tadanobu Takahashi, Tadamune Otsubo, Kiyoshi Ikeda, Takashi Suzuki

**Affiliations:** 1 Department of Biochemistry, School of Pharmaceutical Sciences, University of Shizuoka, Shizuoka, Japan; 2 Department of Organic Chemistry, School of Pharmaceutical Sciences, Hiroshima International University, Hiroshima, Japan; Universidade de São Paulo, BRAZIL

## Abstract

The two main molecular species of sialic acid existing in nature are *N*-acetylneuraminic acid (Neu5Ac) and *N*-glycolylneuraminic acid (Neu5Gc). Neu5Ac is abundant in mammalian brains and plays crucial roles in many neural functions. In contrast, Neu5Gc is present only at a trace level in vertebrate brains. The brain-specific suppression of Neu5Gc synthesis, which is a common feature in mammals, suggests that Neu5Gc has toxicity against brain functions. However, *in vivo* kinetics of Neu5Gc in the whole body, especially in the brain, has not been studied in sufficient detail. To determine the *in vivo* kinetics of Neu5Gc, ^14^C-Neu5Gc was enzymatically synthesized and injected into rat tail veins. Although most of ^14^C-Neu5Gc was excreted in urine, a small amount of ^14^C-Neu5Gc was detected in the brain. Brain autoradiography indicated that ^14^C-Neu5Gc was accumulated predominantly in the hippocampus. ^14^C-Neu5Gc transferred into the brain was incorporated into gangliosides including GM1, GD1a, GD1b, GT1b and GQ1b. Reduction of ^14^C-Neu5Gc after intracerebroventricular infusion was slower than that of ^14^C-Neu5Ac in the brain and hippocampus. The results suggest that Neu5Gc is transferred from blood into the brain across the blood brain barrier and accumulates in the brain more preferentially than does Neu5Ac.

## Introduction

Sialic acid is an acidic monosaccharide and is expressed most frequently at the end of glycan chains to provide a negative charge on the cell surface. The two most prevalent species of sialic acids existing in nature are *N*-acetylneuraminic acid (Neu5Ac) and *N*-glycolylneuraminic acid (Neu5Gc) [1,2,3]. Neu5Ac is abundantly contained in the mammalian brain and plays crucial roles in many neural functions such as memory, synaptic plasticity, brain development and neurological disorder [4,5,6]. In contrast, Neu5Gc is synthesized via the effect of cytidine monophospho-*N*-acetylneuraminic acid hydroxylase (CMAH), which catalyzes the conversion of cytidine 5'-monophosphate Neu5Ac (CMP-Neu5Ac) to CMP-Neu5Gc [7]. Since mRNA of CMAH is not expressed in the mammalian brain, Neu5Gc exists in the brain only at a trace level [7,8,9,10]. Although humans are unable to synthesize Neu5Gc due to a CMAH gene mutation, Neu5Gc expression has been reported in normal human cells and various human cancer cells [1,11,12,13,14]. Neu5Gc is contained in foods, especially in red meat [12,15]. Neu5Gc from dietary sources ingested into humans is incorporated into newly synthesized glycoproteins [11].

Sialidase, which removes sialic acid residues from sialylglycoconjugates, generally cleaves Neu5Ac residues more preferentially than Neu5Gc residues [8,16]. We reported the catalytic preference of Salmonella typhimurium LT2 sialidase for Neu5Ac residues over Neu5Gc residues [17]. Thus, sialyl signalling controlled by sialidase receives a competitive influence of Neu5Gc. Polysialic acid (PSA) plays crucial roles in hippocampal synaptic plasticity, memory and brain development [18,19,20,21,22]. Since α2-8-linked Neu5Gc incorporated into PSA resists sialidase breakdown, it has been assumed that even a small amount of Neu5Gc is harmful for brain functions [8]. However, *in vivo* kinetics of Neu5Gc in the whole body, especially in the brain, remains poorly understood.

To determine the *in vivo* kinetics of Neu5Gc, we synthesized ^14^C-Neu5Gc from ^14^C-sodium pyruvate and *N*-glycolylmannosamine using *N*-acetylneuraminic acid aldolase. The *in vivo* kinetics of ^14^C-Neu5Gc in a rat`s whole body was investigated after tail vein injection and compared to that of ^14^C-Neu5Ac. Since ^14^C-Neu5Gc was detected in the brain after tail vein injection, detailed distribution of ^14^C-Neu5Gc in the brain was imaged using autoradiography. Additionally, since ^14^C-Neu5Gc was accumulated in the brain more preferentially than was ^14^C-Neu5Ac, the *in vivo* kinetics of ^14^C-Neu5Gc after intraventricular injection was compared to that of ^14^C-Neu5Ac.

## Materials and Methods

### Experimental animals

Male Wistar rats (8 weeks old, 166–179 g in b.w.) were purchased from Japan SLC (Shizuoka, Japan). They were housed under standard laboratory conditions (23 ± 1°C, 55 ± 5% humidity) and had access to tap water and diet ad libitum. The lights were automatically turned on at 8:00 and off at 20:00.

### Statement on animal welfare

The animals were cared for in accordance with the guidelines established by the University of Shizuoka. All animal experiments were pre-approved by the Animal Ethics Committee of the University of Shizuoka.

### Synthesis of ^14^C-Neu5Gc

^14^C-Neu5Gc was enzymatically synthesized from 250 μmol sodium pyruvate, 1.85 MBq (or 27.8 MBq for autoradiography) [1-^14^C]-sodium pyruvate (352 MBq/mmol, PerkinElmer) and 300 μmol ManNGc using 1.0 U *N*-acetylneuraminic acid aldolase from Escherichia coli (Nacalai tesque) [23]. The reactants were incubated at 37°C for 48 hours in 200 μl of 20 mM phosphate buffer (pH 7.4). After filtration using an ultrafiltration membrane (30 kDa cut-off, Merck Millipore), the product was applied to a column filled with 50 ml of anion exchange resin (Dowex 1x8, 200–400 mesh, HCOO− form). ManNGc was washed out with 5 bed volumes of water, and then Neu5Gc was eluted with 10 bed volumes of 0.3 M formic acid. The fractions containing Neu5Gc were determined by Ehrlich's reagent and concentrated by a rotary evaporator (Tokyo Rikakikai) and vacuum freeze drying equipment (Tokyo Rikakikai). ^14^C-Neu5Ac was synthesized from ManNAc by incubating for 24 hours and purifying in the same way. The fractions were shown to have a single radioactive peak by assaying with thin layer chromatography (TLC) using propanol/acetic acid/water (60:10:25) as the mobile phase. The yields of ^14^C-Neu5Gc and ^14^C-Neu5Ac were determined by using a liquid scintillation cocktail (Hionic-Fluor, PerkinElmer) and a liquid scintillation counter (LSC) (LSC-3100, Aloka) to be 19.5% and 34.1%, respectively. They were dissolved in 200 mM phosphate buffer and stored at −30°C.

### Measurement of radioactivity in each organ

^14^C-Neu5Gc or ^14^C-Neu5Ac (50 kBq/170 g of body weight) in 200 μl of 200 mM phosphate buffer was injected into rat tail veins. One, 3 or 24 hours after the injection, the rats were anesthetized with chloral hydrate (70 mg/kg). After collection of blood and urine, rats were transcardially perfused with phosphate buffered saline (PBS, pH 7.4) to remove blood from the whole body. Each sample (50–200 mg) of urine, blood and organs including the kidney, liver, lung, spleen, heart, left cerebral hemisphere, right hippocampus and right cerebellar hemisphere was dissolved in 1 ml of SOLVABLE (PerkinElmer) overnight at 50°C, and 0.5 ml of isopropanol and 0.5 ml of hydrogen peroxide were added. After addition of liquid scintillation cocktail (10 ml, Hionic Flour, PerkinElmer), radioactivities were measured using the LSC [24]. Total amounts of radioactivity in each whole organ were calculated from their volume or weights. The total blood volume was estimated from body weight (60 ml/kg B.W.). The total weights of the brain, hippocampus and cerebellum were estimated from hemisphere or unilateral weights.

### Brain autoradiography

Three hours after injection of ^14^C-Neu5Gc (1.0 MBq/170 g of body weight, 200 μl) or ^14^C-Neu5Ac (1.0 MBq/170 g of body weight, 200 μl) into a tail vein, the rats were transcardially perfused with PBS under anesthesia. The brains were removed and used for autoradiography and ganglioside analysis. For the autoradiography [25], the brains were embedded into ice-cold 4% sodium carboxymethyl cellulose and frozen at −30°C. Serial coronal brain sections were cut into 400-μm-thick sections at −15°C with a cryotome (Cryotome CR-502, Nakagawa) and dried at −30°C. After exposure to imaging plates (Storage Phosphor Screen BAS-IP SR 2025E, 20 cm × 25 cm, GE Healthcare) for 2 months, distribution of radioactivity in each section was determined using an autoradiographic scanner (Tyhoon 9400, GE Healthcare).

### Measurement of radioactivity in each ganglioside

Total lipid and protein fractions were separated from whole brain homogenate by the Folch and the Bligh and Dyer (B/D) extraction methods using a monophasic solution of chloroform/methanol/water (C/M/W) [26]. Briefly, the brain was homogenized in water equal to 1.5 times of homogenate volume. The homogenate (0.1 mL) was mixed with 2.25 ml of chloroform/methanol (C/M, 2:1, v/v) and incubated at 37°C for 60 min with constant shaking. After adding 650 μl of methanol, the mixture was centrifuged (3,000 × g) at 4°C for 30 min and the supernatant was removed. The residue was extracted again with 500 μl of C/M/W (5:10:4, v/v/v) at 37°C for 30 min and centrifuged (3,000 × g) at 4°C for 30 min. The two extracts were combined, evaporated with a centrifugal evaporator, and used as the lipid fraction. The residue was used as the chloroform-methanol insoluble fraction including proteins.

For ganglioside analysis, the lipid fractions were redissolved in 5 ml of C/M (9:1, v/v) and applied to a phenyl sepharose column (2 ml, Phenyl Sepharose CL-4B, Sigma-Aldrich) equilibrated with C/M (9:1, v/v) [27]. After elution of most neutral lipids and phospholipids with 10 ml of C/M (9:1, v/v) and 10 ml of C/M (85:15, v/v), gangliosides were eluted with 10 ml of C/M (1:1, v/v) and 10 ml of methanol. Each fraction was redissolved in 50 μl of C/M (2:1, v/v). Gangliosides were separated by high-performance thin layer chromatography (HPTLC, Merck Millipore) using C/M/0.25% CaCl_2_ (50:40:10, v/v/v) and visualized with resorcinol/HCl reagent [28]. The ganglioside spots were scratched and extracted with 200 μl/mg of C/M/W (10:10:1, v/v/v). The radioactivity in each fraction was measured in the same manner as that described above.

### Intraventricular injection

Under anaesthesia, 5 μl of ^14^C-Neu5Gc or ^14^C-Neu5Ac (125 Bq/240 g of body weight) was injected into the right lateral ventricle (AP = −1.0 mm; ML = 1.9 mm; DV = 3.8 mm) [29] through a cannula using a stereotaxic instrument (Narishige). One, 3 or 24 hours after the injection, the rats were transcardially perfused with PBS after blood collection. The radioactivity in the right hippocampus, left hemisphere and blood was measured in the same manner as that described above.

### Statistical analysis

Statistical significance was assessed using a two-tailed unpaired Student’s *t*-test or two-way ANOVA, followed by Bonferroni's multiple comparison test. Statistical analysis was performed using Prism 5 (GraphPad). Error bars are expressed as standard errors of the mean.

## Results

**Fig 1:**
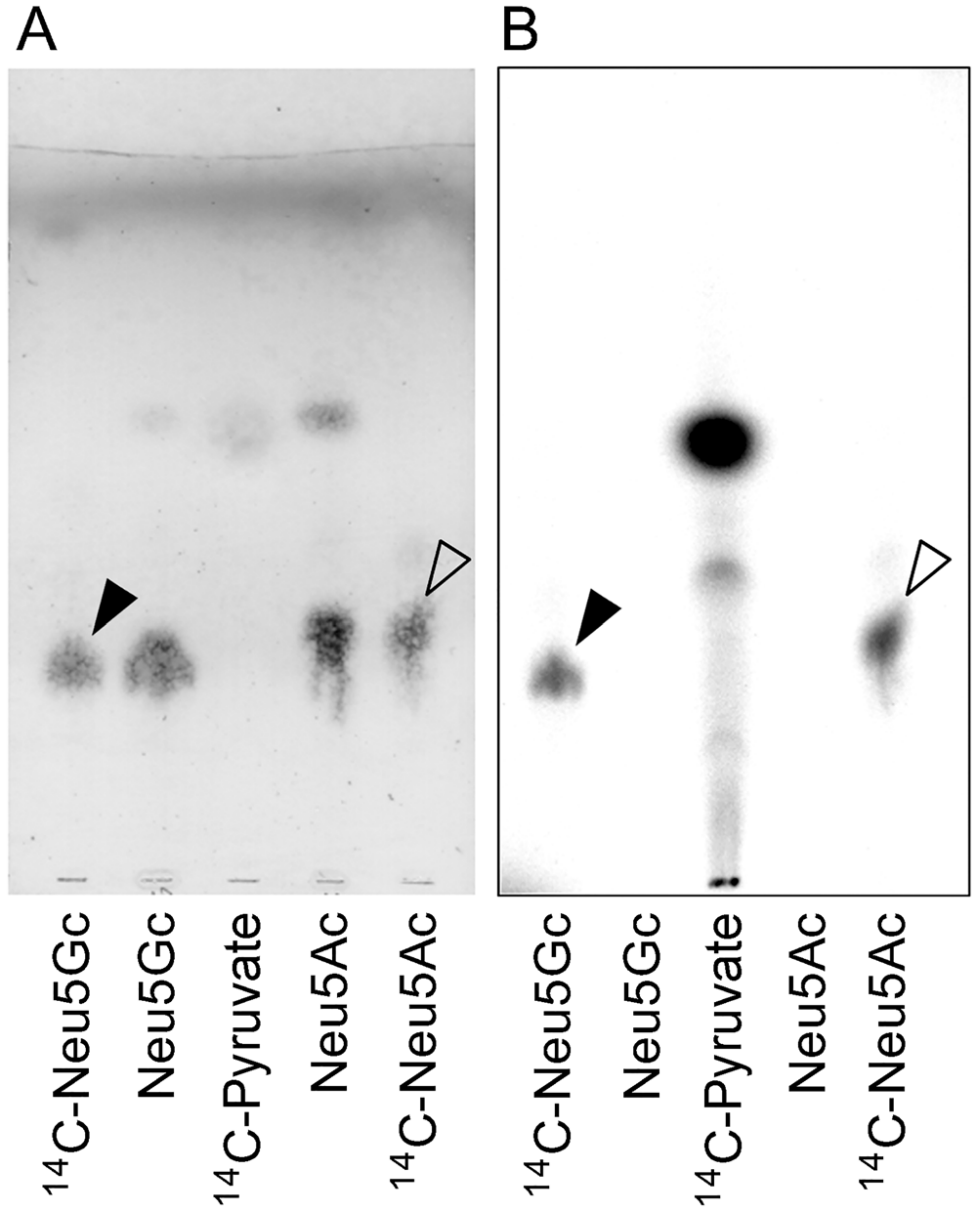
Analysis of synthesized-^14^C-Neu5Gc and ^14^C-Neu5Ac by TLC. After separation by TLC, synthesized ^14^C-Neu5Gc and ^14^C-Neu5Ac were visualized with the orcinol-sulphuric acid reagent (A) and by autoradiography (B). Black-filled and white-filled triangles represent the bands of ^14^C-Neu5Gc and ^14^C-Neu5Ac, respectively.

We synthesized radioisotope-labeled Neu5Gc (^14^C-Neu5Gc) by using *N*-glycolylmannosamine, ^14^C-pyruvic acid and sialic acid aldolase (EC 4.1.3.3, also called sialic acid lyase) under optimum reaction conditions that were determined using unlabeled sodium pyruvate (data not shown). The yield of ^14^C-Neu5Gc was 19.5%, while that of ^14^C-Neu5Ac synthesized using *N*-acetylmannosamine was 34.1%. After purification by an anion exchange resin, single radioactive bands of ^14^C-Neu5Gc and ^14^C-Neu5Ac were detected by autoradiography in TLC (Fig 1A and 1B).

**Fig 2:**
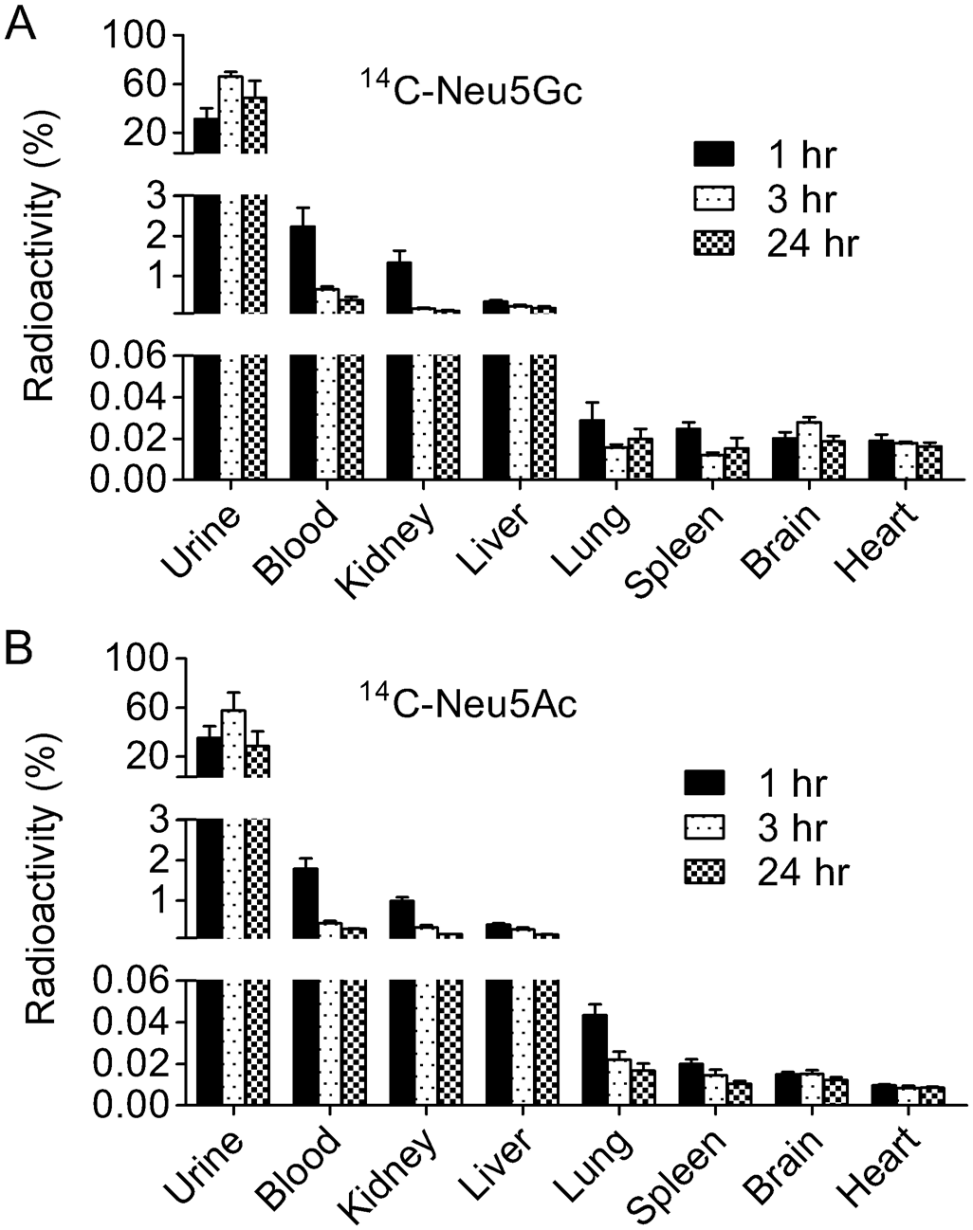
*In vivo* kinetics of ^14^C-Neu5Gc and ^14^C-Neu5Ac in the whole body after tail vein injection. Radioactivity in each organ at 1, 3 and 24 hr after tail vein injection of ^14^C-Neu5Gc (A: *n* = 5) or ^14^C-Neu5Ac (B: *n* = 5–6) is shown as a relative value to the radioactivities of injected-^14^C-Neu5Gc or ^14^C-Neu5Ac.

To determine the *in vivo* kinetics of Neu5Gc in the rat whole body, the distribution of ^14^C-Neu5Gc was investigated after ^14^C-Neu5Gc injection into a tail vein. Most of the radioactivity was detected in urine (Fig 2A). Radioactivity in the blood and organs including the kidney, liver, lung, spleen and heart was detected at 1 hr after ^14^C-Neu5Gc injection and decreased over a period of 24 hr. Radioactivity was also detected in the brain after tail vein injection of ^14^C-Neu5Gc (Fig 2A). The whole body distribution of radioactivity in the ^14^C-Neu5Gc-injected rat was similar to that in the ^14^C-Neu5Ac-injected rat (Fig 2B).

**Fig 3:**
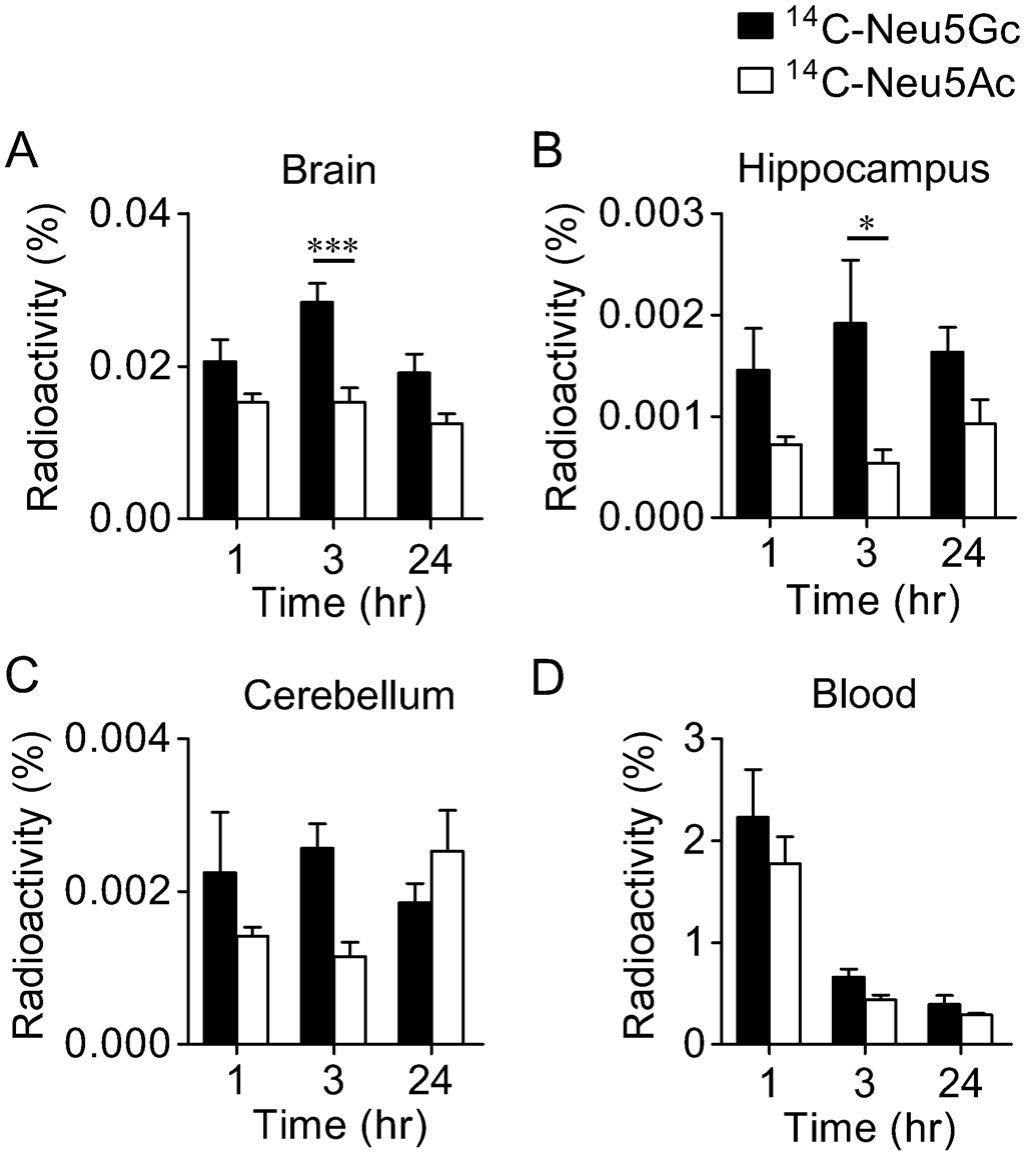
Preferential accumulation of ^14^C-Neu5Gc over ^14^C-Neu5Ac in the brain. Radioactivities in the brain (A), hippocampus (B), cerebellum (C) and blood (D) at 1, 3 and 24 hr after tail vein injection of ^14^C-Neu5Gc (*n* = 5) or ^14^C-Neu5Ac (*n* = 5–6) are shown as relative values to the radioactivities of injected-^14^C-Neu5Gc or ^14^C-Neu5Ac. *P < 0.05 and ***P < 0.001. of ^14^C-Neu5Gc or ^14^C-Neu5Ac.

To investigate the difference between *in vivo* kinetics of Neu5Gc and that of Neu5Ac in the brain, radioactivity of ^14^C-Neu5Gc and that of ^14^C-Neu5Ac were compared after tail vein injection. After ^14^C-Neu5Gc injection, radioactivity in the brain reached a maximum level at 3 hr. In contrast, radioactivity after ^14^C-Neu5Ac injection reached a plateau in less than 1 hr and did not significantly change over a period of 24 hr (Fig 3A). ^14^C-Neu5Gc showed preferential accumulation over ^14^C-Neu5Ac in the brain and hippocampus (Fig 3A and 3B). In the cerebellum and blood, the amounts of ^14^C-Neu5Gc and ^14^C-Neu5Ac were not significantly different (Fig 3C and 3D).

**Fig 4:**
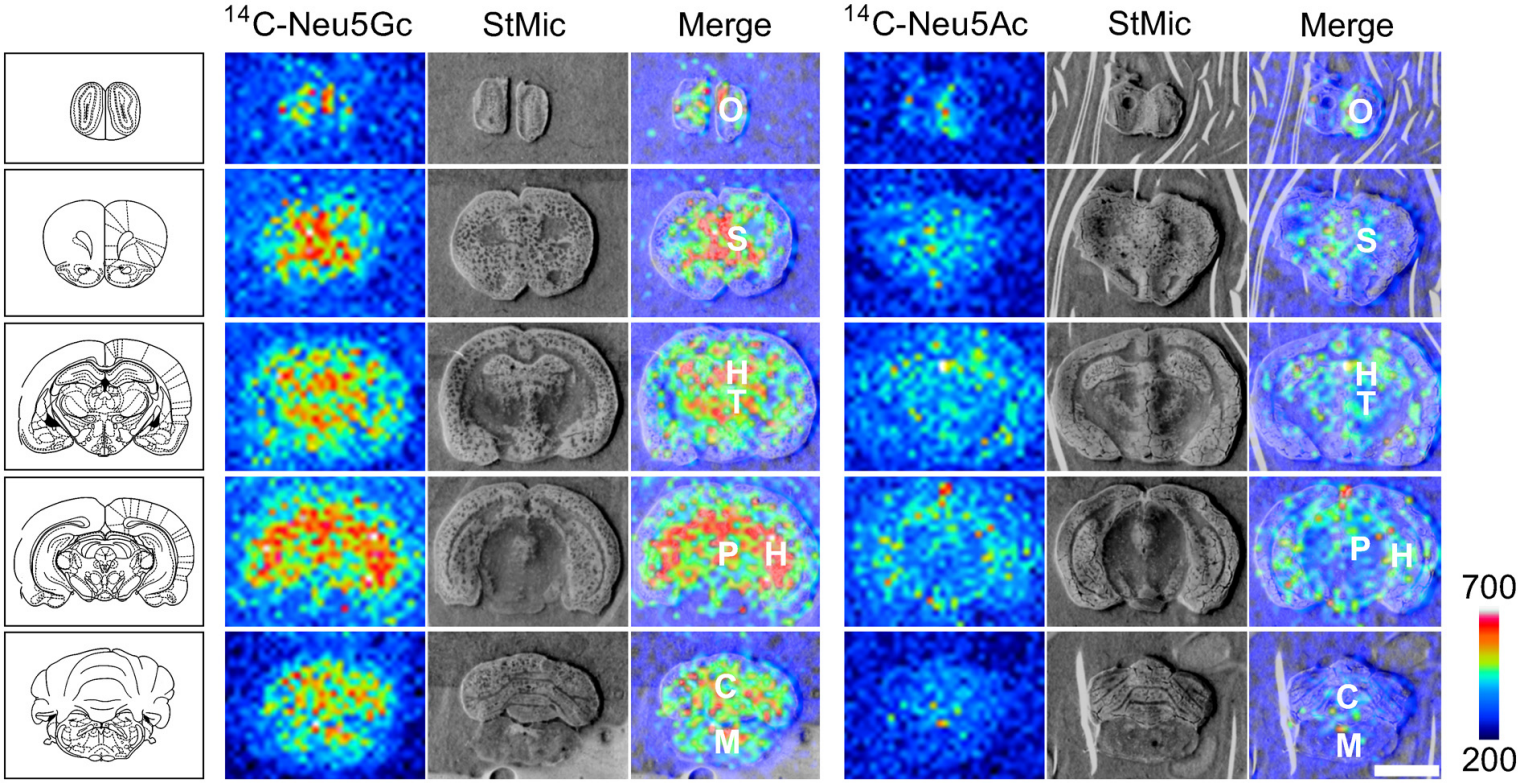
Autoradiograms of ^14^C-Neu5Gc and ^14^C-Neu5Ac in the brain after tail vein injection. Autoradiography of the rat brain was performed at 3 hours after tail vein injection of ^14^C-Neu5Gc (A) or ^14^C-Neu5Ac (B). The experiment was performed twice, and the autoradiograms obtained were almost identical. O, olfactory bulb; S, striatum; H, hippocampus; T, thalamus; P, periaqueductal gray; C, cerebellum; M, medulla. Scale bar, 5 mm.

By using autoradiography, we next determined the detailed distribution of radioactivity in the brain at 3 hr after tail vein injection of ^14^C-Neu5Gc. Although radioactivity was detected from all observed brain regions including the olfactory bulb, striatum, thalamus, periaqueductal gray, cerebellum and medulla, the hippocampus showed intense radioactivity (Fig 4). A similar tendency was obtained by radioactivity quantification. The hippocampus (8.94 ± 3.28 Bq/g tissue) showed higher radioactivity than that of the cerebellum (4.73 ± 0.50 Bq/g tissue) after ^14^C-Neu5Gc injection. In the case of ^14^C-Neu5Ac, radioactivity was also detected from all observed brain regions, but its signal intensity was weak compared to that of ^14^C-Neu5Gc (Fig 4). Radioactivity in the hippocampus (2.76 ± 0.31 Bq/g tissue) after ^14^C-Neu5Ac injection was also higher than that in the cerebellum (2.31 ± 0.31 Bq/g tissue).

**Fig 5:**
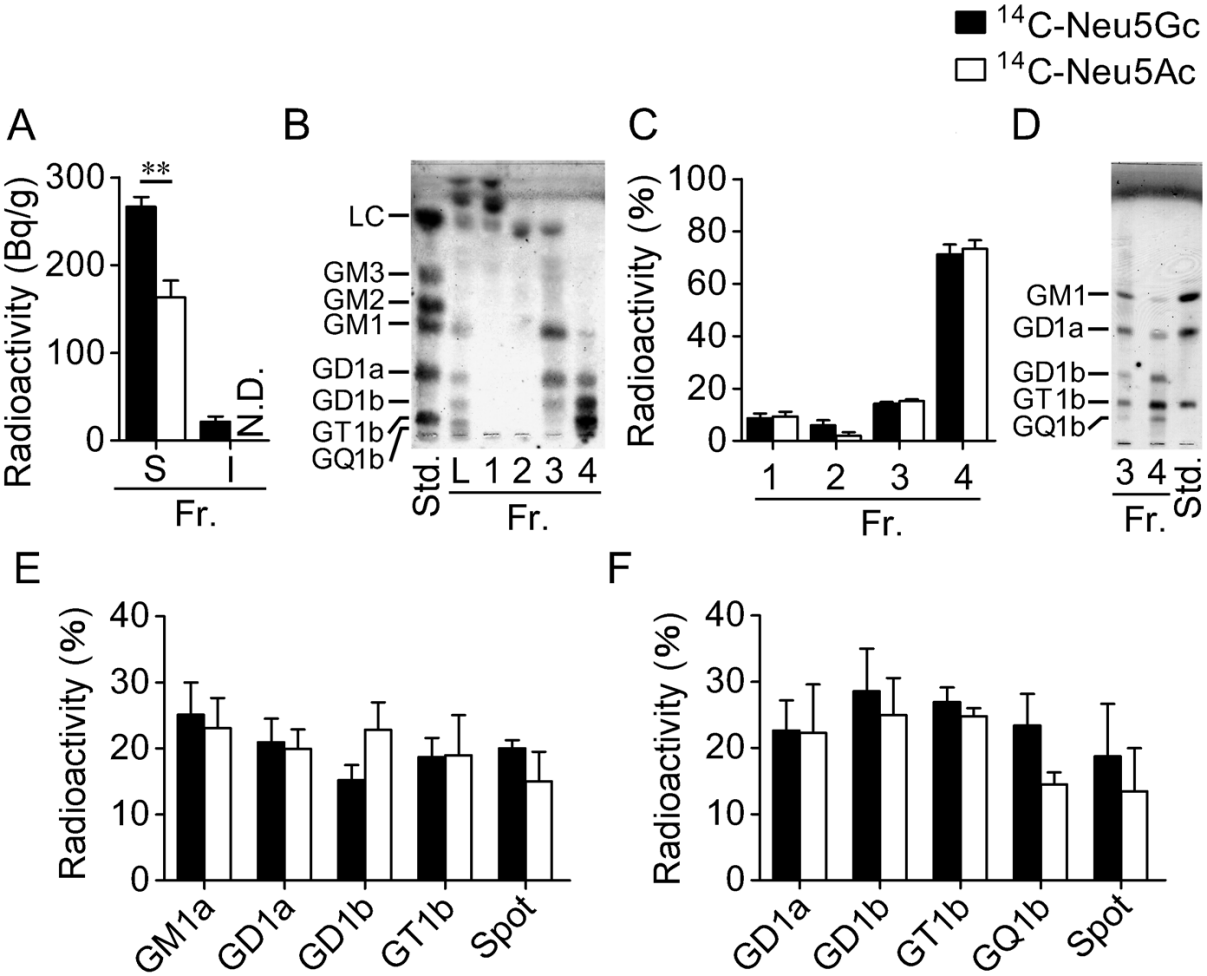
Incorporation of ^14^C-Neu5Gc into gangliosides in the brain after tail vein injection. (A) Three hr after tail vein injection of ^14^C-Neu5Gc (*n* = 3) or ^14^C-Neu5Ac (*n* = 3), chloroform-methanol soluble (S) and insoluble (I) fractions (Fr.) were obtained from the rat brain and radioactivity was measured. N.D., not detected. **P < 0.001. (B) After applying the lipid fraction onto a phenyl sepharose column, the column was washed with C/M (9:1, fr. 1) and C/M (85:15, fr. 2), and then gangliosides were eluted with C/M (1:1, fr. 3) and methanol (fr. 4). Gangliosides in each fraction were analyzed by TLC. (C) The radioactivity in each fraction is shown as a relative value to the total radioactivities in all four fractions (^14^C-Neu5Gc, *n* = 3; ^14^C-Neu5Ac, *n* = 3). (D) Gangliosides were separated by HPTLC using C/M/0.25% CaCl_2_ (50:40:10, v/v/v) and visualized with resorcinol/HCl reagent. (E and F) Radioactivities in each band are shown as relative values to the total radioactivities in fr. 3 (E, *n* = 3) or fr. 4 (F, *n* = 4).

To determine whether ^14^C-Neu5Gc transferred from blood to the brain is used for a component of sialylglycoconjugates such as glycolipids and glycoproteins, we measured radioactivity levels in chloroform-methanol soluble and insoluble fractions derived from the brain after ^14^C-Neu5Gc injection into a tail vein. Although radioactivity was detected in both fractions, the chloroform-methanol soluble fraction (lipid fraction) showed higher radioactivity than that of the chloroform-methanol insoluble fraction including proteins (Fig 5A). In both fractions, radioactivity after ^14^C-Neu5Gc injection was higher than that after ^14^C-Neu5Ac injection.

To obtain direct evidence that ^14^C-Neu5Gc was used as a component of glycolipids, gangliosides in the lipid fraction were separated using hydrophobic interaction chromatography and were analyzed for radioactivity. After applying the lipid fraction onto a phenyl sepharose column, the column was washed with C/M (9:1, fraction 1) and C/M (85:15, fraction 2). After elution with C/M (1:1, fraction 3) and methanol (fraction 4), gangliosides were separated by TLC and detected with resorcinol/HCl reagent. Fractions 3 and 4 contained large amounts of gangliosides, and intense radioactivity was detected (Fig 5B and 5C). Next, gangliosides in fractions 3 and 4 were separated precisely using HPTLC (Fig 5D). As a result of radioactivity measurement in each ganglioside, radioactivity was detected from all of the measured gangliosides including GM1, GD1a, GD1b, GT1b and GQ1b (Fig 5E and 5F). In the case of ^14^C-Neu5Ac injection, radioactivity was also detected from all of the measured gangliosides. Radioactivity derived from proteins in the chloroform-methanol insoluble fraction was also analyzed by Western blotting, but the radioactivity was too low to be detected by autoradiography (data not shown).

**Fig 6:**
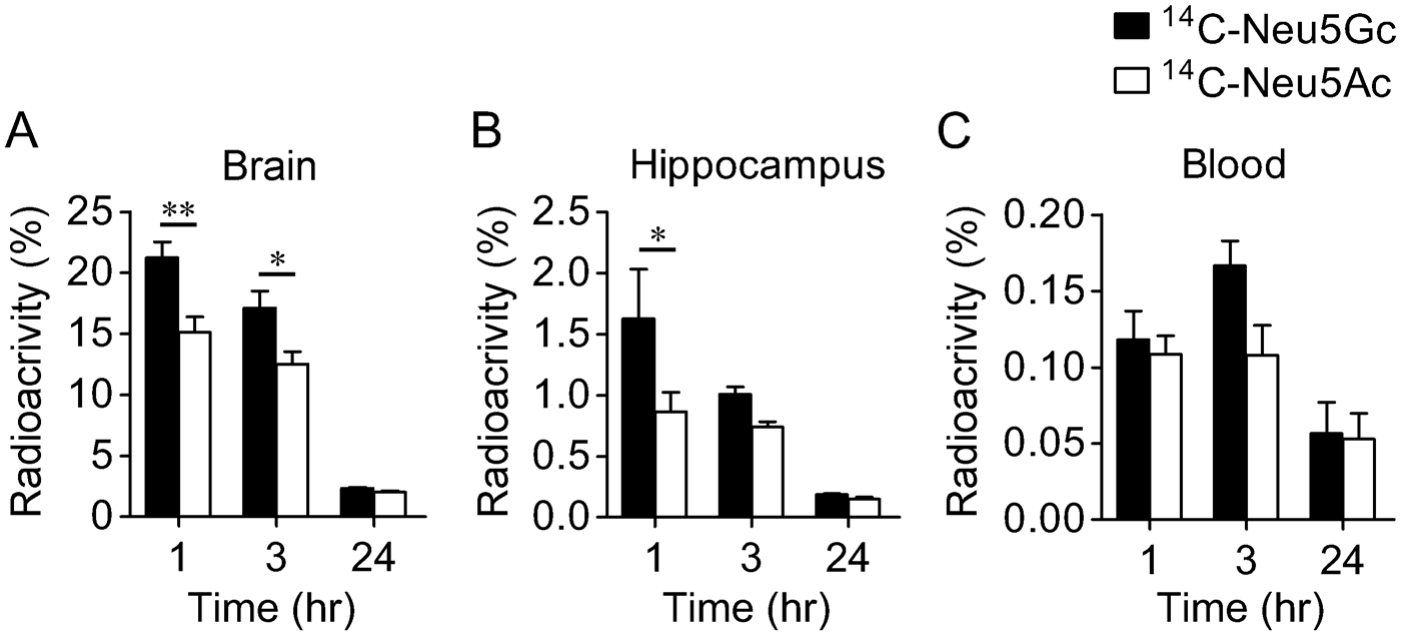
Excretion of ^14^C-Neu5Gc and ^14^C-Neu5Ac after intracerebroventricular injection. Radioactivities in the right brain hemisphere (A), the left hippocampus (B) and blood (C) at 1, 3 and 24 hr after right lateral ventricle injection of ^14^C-Neu5Gc (*n* = 5) or ^14^C-Neu5Ac (*n* = 5) are shown as relative values to the radioactivities of injected-^14^C-Neu5Gc or ^14^C-Neu5Ac. *P < 0.05 and **P < 0.01.

To investigate in more detail the kinetics of Neu5Gc in the brain, the elimination of ^14^C-Neu5Gc was compared to that of ^14^C-Neu5Ac. After intracerebroventricular injection of ^14^C-Neu5Gc and ^14^C-Neu5Ac into the lateral ventricle, radioactivity levels decreased over a period of 24 hr in the brain hemisphere and hippocampus (Fig 6A and 6B). Radioactivity levels in the brain hemisphere and hippocampus after ^14^C-Neu5Gc injection were significantly higher than those after ^14^C-Neu5Ac injection. Radioactivity was also detected in the blood after intracerebroventricular injection of ^14^C-Neu5Gc and ^14^C-Neu5Ac (Fig 6C).

## Discussion

The *in vivo* kinetics of Neu5Gc in the rat whole body was investigated using synthesized-^14^C-Neu5Gc. Although most of ^14^C-Neu5Gc was excreted in urine after tail vein injection, a small amount of ^14^C-Neu5Gc was detected in the brain. This result indicates that Neu5Gc is transferred into the brain across the blood brain barrier. ^14^C-Neu5Gc was also detected in other organs including the kidney, liver, lung, spleen and heart. The *in vivo* kinetics of ^14^C-Neu5Gc in the rat whole body except for the brain was approximately consistent with the results of a previous study [30].

Mammalian *N*-acetylneuraminic acid aldolase cleaves not only Neu5Ac but also Neu5Gc [31]. To exclude the possibility that radioactivity detected in the brain was caused by the ^14^C-Neu5Gc cleavage product produced by endogenous enzymes such as *N*-acetylneuraminic acid aldolase, we analyzed the radioactivity in sialylglycoconjugates in brains after tail vein injection of ^14^C-Neu5Gc. Since radioactivity was detected in both chloroform-methanol soluble and insoluble fractions, ^14^C-Neu5Gc would be contained in the brains as components of glycolipids and glycoproteins. Radioactivity was much higher in the lipid fraction than in the chloroform-methanol insoluble fraction including proteins, indicating that Neu5Gc was preferentially used in glycolipids. Results of radioactivity measurements in gangliosides contained in the lipid fraction showed that GM1, GD1a, GD1b, GT1b and GQ1b had radioactivity. Because free Neu5Gc is taken up by mammalian cells via pinocytosis and is metabolically incorporated into glycoconjugates [32], Neu5Gc transferred to the brain would also be taken up by cells and incorporated into gangliosides.

We also compared the accumulation of Neu5Gc with that of Neu5Ac in brain after tail vein injection. ^14^C-Neu5Gc accumulated in the brain and hippocampus more abundantly than did Neu5Ac. Abundant accumulation of Neu5Gc in the brain was also confirmed by using autoradiography. Because the reduction of ^14^C-Neu5Gc in the brain and hippocampus was slower than that of ^14^C-Neu5Ac after intracerebroventricular infusion, abundant accumulation of Neu5Gc would be caused by the slow elimination of Neu5Gc from the brain and hippocampus. The rate of hydrolysis of Neu5Gc by sialidase and *N*-acetylneuraminic acid aldolase is slower than that of Neu5Ac. Actually, the amounts of ^14^C-Neu5Gc in lipid and protein fractions were larger than those of ^14^C-Neu5Ac. Thus, one reason for the persistence of Neu5Gc may be slow elimination by, for example, sialidases. To obtain a better understanding of the different kinetics of Neu5Gc and Neu5Ac, comparison of the catabolic, anabolic and transport efficiency in the brain will be needed.

The brain-specific suppression of Neu5Gc expression, which is a common feature in mammals, may suggest that Neu5Gc has toxicity against brain functions. Our brain imaging of ^14^C-Neu5Gc after tail vein injection by autoradiography indicated that Neu5Gc transferred from blood is highly accumulated in the hippocampus. In the hippocampus, it has been reported that sialyl signalling via gangliosides such as GQ1b and PSA, which bind to neural cell adhesion molecule (NCAM), is necessary for synaptic plasticity and memory [33,34,35]. Sialidase also plays important roles in many neural functions, including neural excitation, axonal elongation, differentiation and maturation [5,36]. Four types of mammalian sialidase (Neu1, Neu2, Neu3 and Neu4) have been identified in mammalian tissues by their localization and enzymatic properties. Shiozaki et al. reported that Neu4 mRNA was expressed abundantly in the hippocampus [37]. We previously found by using a highly sensitive fluorescent histochemical method that the hippocampus, especially in the regions where mossy fiber terminals exist, showed intense sialidase activities [38,39]. Since the cleavage of Neu5Gc by sialidase is much less than that of Neu5Ac, competitive inhibition of sialidase with Neu5Gc may disrupt the regulation of sialyl signalling in the hippocampus.
